# Crisis support teams’ technological openness and learning attitudes toward the AI based virtual patient system *crisis support VR*

**DOI:** 10.3389/fdgth.2026.1805454

**Published:** 2026-06-09

**Authors:** Sophie Kjerstin Mårtensson, Charlotte Hveem, Maurice Lamb, Rajna Knez

**Affiliations:** 1 School of Health Sciences, University of Skövde, Skövde, Sweden; 2 Department of Security, Sahlgrenska University Hospital, Gothenburg, Sweden; 3 School of Informatics, University of Skövde, Skövde, Sweden; 4 Child and Adolescent Medicine, Child and Adolescent Psychiatry and Women's Health, Skaraborg Hospital, Skövde, Sweden; 5 Institute of Neuroscience and Physiology, Sahlgrenska Academy, University of Gothenburg, Gothenburg, Sweden

**Keywords:** digital learning, humanitarian crises, psychological first aid, virtual patient simulation, virtual reality

## Abstract

**Background:**

Against the backdrop of escalating global humanitarian crises, innovative didactic simulations are becoming increasingly important. A promising alternative to traditional classroom-based didactics for learning psychological first aid (PFA) prior to humanitarian crises is to use generative artificial intelligence (GenAI)-based virtual patient (VP) systems. However, there is limited research on the use of GenAI-based VP systems for crisis support teams’ learning. The aim of this study was to describe crisis support teams’ technological openness and learning attitudes toward the GenAI-based VP system called *Crisis Support Virtual Reality* (VR).

**Method:**

This study employed an action research design, originating from the collaboration with the innovation project *Crisis Support VR* in the Västra Götaland region of western Sweden. A total of 32 participants completed the Technological Openness and Learning Attitudes (TOLA) survey. Quantitative data were analyzed using descriptive statistics, while qualitative responses were employed as illustrative quotes.

**Results:**

The participants felt generally positive about practicing PFA for serious events/humanitarian crises using the *Crisis support VR* and believe that it is a good complement to practicing PFA. Perceived learning benefits for participants through observing the avataŕs facial and body expressions in applied GenAI-based VP system received lowest score among all items.

**Discussion:**

*Crisis Support VR* represents an innovative didactic simulation tool for learning PFA, serving as a valuable complement to traditional classroom-based didactics. Future research should evaluate its effects on learning outcomes and, in doing so, contribute to preparedness and response capacity during humanitarian crises.

## Introduction

In today's international research landscape, significant advances are being made in the field of generative artificial intelligence (GenAI)-based virtual patient (VP) systems. These GenAI systems facilitate a variety of learning didactics in realistic learning environments, facilitating the development of communication, decision-making, and psychomotor skills. At the same time, against the backdrop of escalating global humanitarian crises, there is an urgent need to strengthen awareness of and support for the vital role of humanitarian action. The years ahead look bleak: Entrenched and escalating violent conflicts, along with widespread violations of international humanitarian law and ongoing global climate crises, continue to inflict devastating harm on civilian populations.

## Background

The growing complexity of humanitarian crises poses considerable challenges to societal well-being (1). According to the World Health Organization's (WHO's) Mental Health Gap Action Program Humanitarian Intervention Guide (2), a core component of all humanitarian responses is the deployment of crisis support teams trained in psychological first aid (PFA) (3), guided by the five essential principles described by Hobfoll et al. (4): *A sense of safety* refers to the assurance of physical and emotional security for affected individuals; *calming* involves the provision of accurate information and, when needed, medical intervention to alleviate pain; *self- and community efficacy* means fostering confidence in both individual and collective capacity to manage adversity; *social connectedness* encourages the involvement of family and community in reinforcing social bonds; and *hope* seeks to promote and instill a sense of optimism about the future. Hobfoll et al. (4) emphasized that these five principles neither support nor oppose other crisis support methods. They were developed with the aim of being adaptable to specific situations and contexts.

Learning to apply these five principles effectively is essential for ensuring humanitarian responses and, by extension, long-term societal well-being (3). However, the existing variety of learning didactics for PFA are often resource intensive and rely heavily on traditional classroom-based didactics supplemented by didactic simulations. Such learning didactics typically involve synchronous learning sessions, which may vary significantly in quality depending on the expertise of instructors and the dynamics of participant groups (5). As a promising alternative, GenAI-based VP systems have emerged as innovative tools that incorporate didactic simulation and provide structured and repeatable interactions through computer-based platforms (6, 7). Empirical studies (8, 9) have shown that GenAI-based VP systems can facilitate learning outcomes comparable to, or even exceeding, those of traditional classroom-based didactics.

One possible explanation for this is that GenAI-based VP systems facilitate experiential learning in a safe learning environment, enabling learners to develop intuitive and tacit knowledge without the risk of harming real people (10). As can be understood through the theoretical framework of experiential learning, rooted in the work of Dewey (11, 12). Experiential learning emphasizes the integration of past and present experiences to inform future practice. This theoretical framework is further elaborated by Kolb's (13) experiential learning theory, which outlines four interconnected stages: C*oncrete experience* involves direct engagement in a task; *reflective observation* refers to analyzing experiences from multiple viewpoints; *abstract conceptualization* entails integrating the reflections into a broader theoretical understanding; and *active experimentation* involves applying this knowledge in practice to facilitate the development of communication, decision-making, and psychomotor skills (13).

However, the integration of GenAI-based VP systems into humanitarian crisis response learning poses several challenges, such as restrictions on the simulation of humanitarian crisis scenarios, often due to ethical considerations or organizational policy constraints (6, 14). Additionally, educators in the field of humanitarian crises have raised concerns about the perceived lack of authenticity in simulations generated by GenAI-based VP systems (15). Such concerns can affect learners’ engagement and diminish the perceived relevance of the learning content and outcomes (15, 16). These limitations highlight the importance of continuously evaluating GenAI-based VP systems to ensure their authenticity and learning outcomes of these systems in facilitation within the context of humanitarian crises. Thus, the aim of the present study was to describe crisis support teams’ technological openness and learning attitudes toward a GenAI-based VP system called *Crisis Support Virtual Reality* (VR).

## Materials and methods

### Study design

This study, which emerged from collaboration with the innovation project Crisis Support VR in the Västra Götaland Region (VGR) of western Sweden, was based on action research (17, 18). Data were collected through the Technological Openness and Learning Attitudes (TOLA) survey. Quantitative data were analyzed using descriptive statistics (19), while qualitative responses were used as illustrative quotes.

### Context

In the VGR, crisis support teams’ activities in response to serious events depend on the expertise of professionals from multiple disciplines. A serious event is defined as “an event so serious that resources should be organized, managed, and used in a special way” (20). According to the Swedish National Board of Health and Welfare's regulations and general guidelines, regional health and medical care organizations must plan for the deployment of crisis support teams to support individuals who have experienced (or are at risk of experiencing) mental health issues due to a serious event, such as a humanitarian crisis. In the VGR, crisis support is provided by 17 hospitals organized into five hospital groups, along with publicly operated primary care services. Crisis support staff who carry out their regular professional duties under normal circumstances are mobilized as crisis support team members when needed. Currently, approximately 300 staff members are trained and available to participate in crisis support teams across the VGR. Each crisis support team is led by a designated team leader and coordinated at the regional level by a crisis support coordinator based at Sahlgrenska University Hospital (the largest hospital in VGR, located in Gothenburg). Notably, in the VGR, the coauthor of this study (CH) serves as the appointed regional coordinator for crisis support and as the innovation project leader for *Crisis Support VR*.

### Crisis support VR

In the *Crisis Support VR system*, PFA is practiced via GenAI-powered avatars that have experienced serious events/humanitarian crises. *Crisis Support VR* was developed by VirtualSpeech, which provides soft skills training in VR based on AI feedback (https://virtualspeech.com/). Learners who use the *Crisis Support VR* system can select from nine different GenAI-powered avatars that represent diverse functional abilities, ethnic backgrounds, sexes, and gender expressions. One avatar is notably younger than the others, representing a teenager or young adult. A common denominator among all avatars is that they can simulate the response of verbal content, paraverbal communication and nonverbal cues of individuals who have experienced serious events/humanitarian, such as terrorist attacks, floods, or wildfires. Conversations with GenAI-powered avatars take place within various extended reality (XR) environments and can be conducted in 16 different languages (including Swedish, English, Spanish, and Arabic). At the beginning of a learning session, each learner is prompted to select a VR and/or XR environment, a specific serious event/humanitarian crisis, and a GenAI-powered avatar. The subsequent conversation is then based on these selections. After the learner completes the session, the system provides both oral and written GenAI-supported feedback, highlighting the learner's verbal and nonverbal strengths and areas for improvement. This feedback is structured to align with the five essential principles outlined by Hobfoll et al. (4), and it includes a performance rating scale of 1–10, with 1 indicating low and 10 indicating very good. Session data can be saved to facilitate longitudinal tracking of the learner's progress. Following the conversation with the GenAI-powered avatar, the learner is offered a debriefing session with a separate GenAI-powered avatar that has monitored the session, providing an additional reflective learning opportunity.

### The TOLA survey

As no validated survey instruments were identified as suitable for addressing various aspects of technological openness and learning attitudes toward using digital technology, the TOLA survey was developed by the author SM, with some of the items formulated in consultation with author ML. The TOLA survey, are theoretically informed by Kolb's (13) experiential learning theory, the technology acceptance model (TAM) (21), and the unified theory of acceptance and use of technology (UTAUT) model (22). Table 1 presents how each survey item presented in this study is linked to Kolb's (13) learning process; Concrete Experience (CE), Reflective Observation (RO), Abstract Conceptualization (AC), Active Experimentation (AE); TAM (21), including Perceived Usefulness (PU) and Perceived Ease of Use (PEU) (21), and the key constructs of UTAUT (22); Performance Expectancy (PE), Effort Expectancy (EE), Social Influence (SI) Facilitating Conditions (FC).

**Table 1:** The TOLA survey items linked to kolb/TAM/UTAUT.

*Items*	*Kolb/TAM/UTAUT*
1.1. I have good digital competence.	-/-/FC
5.1. I am positive about learning serious events/humanitarian crises with the GenAI based VP system *Crisis Support VR*.	CE/PU/PE
5.2. I believe that *Crisis Support VR* is a good complement to practice psychological first aid.	AC/PU/PE
5.3. I expect that I can learn more about serious events/humanitarian crises with *Crisis Support VR* than through traditional classroom teaching (i.e., meetings between educators and learners).	AC/PU/PE
5.4. I want to continue practicing psychological first aid with *Crisis Support VR*.	AE/PU/PE
5.5. I believe that the digital technology in the *Crisis Support VR* system worked well.	-/PEU/EE
5.6. I believe it was easy to understand the instructions about navigating *Crisis Support VR*.	-/PEU/EE
5.7. I believe it was easy to navigate *Crisis Support VR*.	-/PEU/EE
5.8. I believe that *Crisis Support VR* represented a variety of crisis situations (i.e., terrorist attacks, wildfires, floods, etc.).	CE, AE/PU/PE
5.9. I believe that *Crisis Support VR* reflects a norm-critical perspective, meaning that it is designed to minimize built-in biases.	AC/PU/PE
5.10. I believe that *Crisis Support VR* gave me relevant feedback on my performance.	RO/PU/PE
5.11. I felt safe when I practiced psychological first aid with *Crisis Support VR*.	RO/PU/PE
5.12. I believe that in *Crisis Support VR* I could practice listening to the avatar.	CE, AE/PU/PE
5.13. I believe that in *Crisis Support VR* I could practice seeing the avatar's facial and body expressions.	CE, AE/PU/PE
5.14. I believe that in *Crisis Support VR* I could practice asking questions to the avatar.	CE, AE/PU/PE

To assess face validity, the survey items were pilot tested with one healthcare professional specialising in crisis support, two experts in information technology, and three undergraduate nursing students. The TOLA survey required approximately 10 to 15 min to complete. Feedback from the pilot testing resulted in minor revisions and clarifications of wording across the five thematic sections of the survey. These adjustments were made to ensure that the items and statement were clearly formulated, concise, and free from ambiguity, thereby facilitating consistent interpretation among respondents. The final TOLA survey included six items regarding demographic characteristics and five thematic sections (see the appendix). Each thematic section contains statements rated on a five-point Likert scale, with 1 indicating “Not at all true” and 5 indicating “Completely true.” Two themes also include open-ended questions that allow for free-text responses.

The complete list of the TOLA survey items and statements is provided in the appendix and forms part of a larger umbrella project (results not yet published) examining technological openness and learning attitudes toward using digital technology among healthcare and rescue service professionals as well as higher education healthcare and information technology students. Within the umbrella project, larger samples are planned to enable comprehensive psychometric validation, including reliability metrics, to strengthen the measurement properties of the TOLA survey.

### Data collection

**Figure 1:**
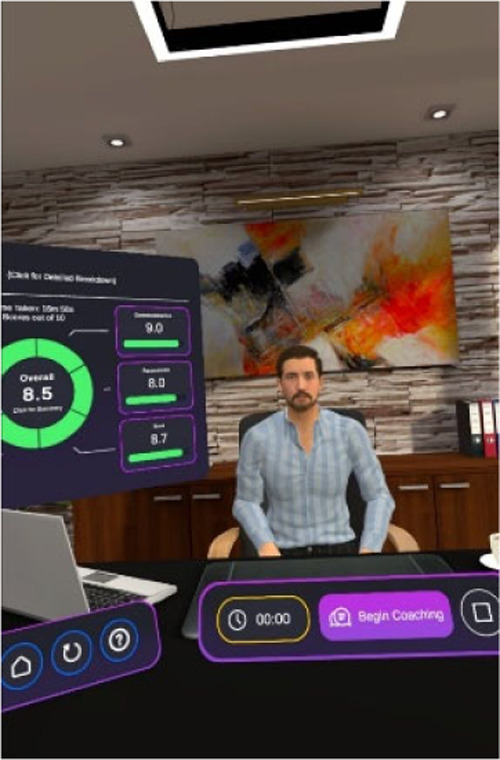
VIVE Focus 3.

**Figure 2:**
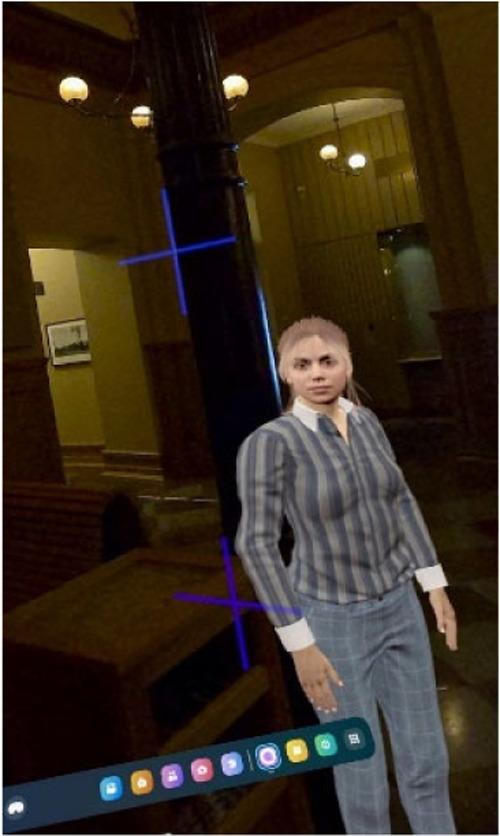
Meta Quest 3.

To demonstrate the GenAI-based *Crisis Support VR system*, the coauthor of this article (CH), in her role as regional coordinator for crisis support**,** invited 173 professionals from various professions in the VGR to participate in this project by completing the TOLA survey during the winter and spring of 2025. CH led each demonstration session, which began with a 30-minute lecture outlining the organization and mission of the crisis support team, the five essential principles described by Hobfoll et al. (4), and an introduction to the *Crisis Support VR system*. During each demonstration session, two different models of VR headsets were made available: VIVE Focus 3 (Figure 1) and Meta Quest 3 (Figure 2). VIVE Focus 3 offers a more advanced VR experience with higher resolution and a wider field of view. In contrast, Meta Quest 3 has mixed-reality capabilities, which allow learners to visualize real-world environments with their virtual GenAI-powered avatars overlaid and activated by pressing the side of the headset. Following completion of the *Crisis Support VR* practice sessions, CH debriefed the participants who had engaged voluntarily in the *Crisis Support VR* practice during the sessions; thus, not all attendees chose to try out the VR system.

Of the 173 professionals invited to participate, 108 responded to the TOLA survey. Of these, 57 completed the thematic section focusing on *Crisis Support VR*. Four respondents were excluded based on free-text comments indicating that they had not practiced with the *Crisis Support VR*, resulting in a final sample of 53 participants. The main demographic characteristics of the participants (sex, age, and profession) are presented in Table 2.

**Table 2:** Participants’ demographic characteristics (*N* = 53).

Characteristics	Number (%)
Sex	
**Female**	34 (64.2)
**Male**	19 (35.8)
Age (years)	
**26–35**	15 (28.3)
**36–45**	13 (24.5)
**46–55**	14 (26.4)
**56–65**	11 (20.8)
Profession	
**Physician**	24 (45.3)
**Psychologist or counselor**	8 (15.1)
**Rescue service professional**	8 (15.1)
**Nurse**	6 (11.3)
**Other (e.g., assistant nurse or administrative assistant)**	7 (13.2)

Based on their responses, the participants’ professional experience ranged from 1 to 34 years (mean = 12.91). Participants who did not respond to all survey items were not excluded; therefore, the number of responses varied across items, ranging from 36 to 53 (Table 3). Participation in the study was voluntary and confidential, and participants were informed that they could withdraw from the study at any time without providing a reason.

**Table 3:** Crisis support teams’ technological openness and learning attitudes toward the GenAI-based VP system, *crisis support VR.*

Items	Number of answers	Mean	1 Not at all true	2	3	4	5 Completely true
**1.1. I have good digital competence.**	53	3.85	1	1	16	22	13
**5.1. I am positive about learning serious events/humanitarian crises with the GenAI based VP system *Crisis Support VR*.**	53	4.15	4	2	7	9	31
**5.2. I believe that *Crisis Support VR* is a good complement to practice psychological first aid.**	45	4.27	1	4	4	9	27
**5.3. I expect that I can learn more about serious events/humanitarian crises with *Crisis Support VR* than through traditional classroom teaching (i.e., meetings between educators and learners).**	46	3.52	4	4	15	10	13
**5.4. I want to continue practicing psychological first aid with *Crisis Support VR*.**	38	4.16	3	0	5	10	20
**5.5. I believe that the digital technology in the *Crisis Support VR* system worked well.**	43	3.84	1	3	11	15	13
**5.6. I believe it was easy to understand the instructions about navigating *Crisis Support VR*.**	41	3.83	2	3	9	13	14
**5.7.** I believe it was easy to navigate *Crisis Support VR*.	38	3.66	2	4	10	11	11
**5.8.** I believe that *Crisis Support VR* represented a variety of crisis situations (i.e., terrorist attacks, wildfires, floods, etc.).	36	3.67	1	3	10	15	7
**5.9.** I believe that *Crisis Support VR* reflects a norm-critical perspective, meaning that it is designed to minimize built-in biases.	37	3.43	1	4	17	8	7
**5.10. I believe that *Crisis Support VR* gave me relevant feedback on my performance.**	39	3.87	2	1	9	15	12
**5.11. I felt safe when I practiced psychological first aid with *Crisis Support VR*.**	38	4.05	1	1	8	13	15
**5.12. I believe that in *Crisis Support VR* I could practice listening to the avatar.**	39	3.82	3	1	8	15	12
**5.13. I believe that in *Crisis Support VR* I could practice seeing the avatar's facial and body expressions.**	39	3.00	4	8	15	8	4
**5.14.** I believe that in *Crisis Support VR* I could practice asking questions to the avatar.	38	3.95	3	0	7	14	14

GenAI-based VP system, generative artificial intelligence (GenAI)-based virtual patient (VP) systems; Crisis Support VR, Crisis Support Virtual Reality (VR).

### Data analysis

For this study, variables were selected from the fifth thematic section of the TOLA survey that applied to *Crisis Support VR* (statements 5.1–5.14.), as presented in Table 3, along with the open-ended question, “Would you like to share any additional thoughts about how you perceive your learning with *Crisis Support VR*?” In addition, the statement from the 1.1 thematic section “I have good digital competence” was included in the analysis as a reference measure of the participants’ self-evaluated digital competence (Table 4).

**Table 4:** Crisis support teams’ technological openness and learning attitudes toward the GenAI-based VP system, *crisis support VR* (*N* = 32).

Items	Mean
**1.1. I have good digital competence.**	3.72
**5.1. I am positive about learning serious events/humanitarian crises with the GenAI-based VP system *Crisis Support VR*.**	4.28
**5.2. I believe that *Crisis Support VR* is a good complement to practice psychological first aid.**	4.25
**5.3. I expect that I can learn more about serious events/humanitarian crises with *Crisis Support VR* than through traditional classroom teaching (i.e., meetings between educators and learners).**	3.56
**5.4** I want to continue practicing psychological first aid with *Crisis Support VR*.	4.09
**5.5** I believe that the digital technology in the *Crisis Support VR* system worked well.	3.84
**5.6** I believe it was easy to understand the instructions on how to navigate *Crisis Support VR.*	3.81
**5.7** I believe it was easy to navigate *Crisis Support VR*.	3.63
**5.8** I believe that *Crisis Support VR* represented a variety of crisis situations (i.e., terrorist attacks, wildfires, floods, etc.).	3.72
**5.9** I believe that *Crisis Support VR* reflects a norm-critical perspective, meaning it is designed to minimize built-in biases.	3.53
**5.10. I believe that *Crisis Support VR* gave me relevant feedback on my performance.**	3.84
**5.11. I felt safe when I practiced psychological first aid with *Crisis Support VR*.**	4.03
**5.12. I believe that in *Crisis Support VR* I could practice listening to the avatar.**	3.72
**5.13. I believe that in *Crisis Support VR* I could practice seeing the avatar's facial and body expressions.**	2.97
**5.14.** I believe that in *Crisis Support VR* I could practice asking questions to the avatar.	3.91

GenAI-based VP system, generative artificial intelligence (GenAI)-based virtual patient (VP) systems; Crisis Support VR, Crisis Support Virtual Reality (VR).

Descriptive statistics were employed to present the quantitative results. Likert scale scores of 4 and 5 represented a high level of agreement or self-evaluated competence, a score of 3 indicated moderate agreement/competence, and scores of 1 and 2 reflected low agreement/competence. Qualitative data derived from responses to the open-ended questions are presented as illustrative quotes in this article to support the quantitative results.

## Results

The results from the part of the TOLA survey that addresses *Crisis Support VR*, including means for all the statement variables related to crisis support teams’ technological openness and learning attitudes toward the GenAI-based VP system, as well as response frequencies for the Likert scale, are presented in Table 3.

A total of 32 participants completed all statements in the TOLA survey—Crisis Support section, with an overall mean score of 3.79 (statements 1.1., 5.1–5.14. Table 4).

Across all professional groups, 51 of 53 participants (96%) rated their self-evaluated digital competence (statement 1.1.) as sufficiently good (scores of 3–5; see Table 3), and among them, 35 rated their digital competence as high (scores of 4 and 5). The physicians, who constituted the most represented group in our sample, all reported moderate or high digital competence, with 20 out of the 24 reporting high digital competence and, eight reporting the maximum level of digital competence (scores of 5). Among the psychologists and counselors, seven out of eight participants reported moderate or high digital competence. All the rescue service professionals reported moderate or high digital competence. Five out of six nurses reported a moderate level of self-evaluated digital competence.

The participants felt generally positive about practicing PFA for serious events/humanitarian crises using the GenAI-based VP system *Crisis Support VR* (mean = 4.15, Table 3). Notably, among participants completed the whole survey, the highest-rated statements in the entire survey related to positive feelings towards learning serious events/humanitarian crises with the GenAI-based VP system *Crisis Support VR* (statement 5.1.; mean = 4.28, Table 4) and the belief that *Crisis Support VR* can complement for practicing PFA (statement 5.2.), which received a mean score of 4.25 (Table 4). Qualitative data gathered from the open-ended questions further contextualized these results. Although some participants had positive experiences (“It was fun … and it was good training”)*,* others expressed reservations (“I am cautiously positive”), suggesting the *Crisis Support VR* system has potential but there is a need for further development and refinement of the *Crisis Support VR* system.

Among the statements 5.5–5.7. the one concerning the ease of navigating *Crisis Support VR* received the lowest score (mean = 3.63; Table 4). Qualitative responses suggested that generational differences may have influenced perceptions of the system's usability, as expressed by one participant: “If you are from my generation and have not practiced hand control movements and simultaneously using the arrows to answer questions, you can lose concentration easily. This was not good for our conversation, but it can only get better.” This statement emphasizes potential challenges related to motor coordination and interface familiarity for learners and highlights areas for improving the system's accessibility and design.

Among the statements 5.8–5.11. the statement concerning the *Crisis Support VR* system's norm-critical perspective received the lowest mean score (mean = 3.53, Table 4), while the statement concerning participants’ sense of safety during practice received the highest score (mean = 4.03, Table 4). The qualitative responses provided further insight into these quantitative values. Several participants expressed appreciation for the opportunity to engage in repeated practice, with comments such as “What I liked most was the opportunity for volume training” and “Good for volume training.” However, some participants also suggested the need for greater scenario variation to enhance learning, with one participant stating, “With a few more varied scenarios, one would get the opportunity for better training*”* and another anticipating future system improvements: “It is going to be exciting when it becomes possible to practice with a suicidal person [avatar], and I hope that in the future it will be possible to practice conversations with a group, and that two people could lead the conversation. Fantastic support tools.”

In addition, some participants commented on the response time in the conversation with the avatar as an important system feature, which could be perceived as somewhat strange but still tolerable, as indicated by the response: “I think it feels a bit stiff at times when talking with the avatar. But overall, it was cool to get to test it.” Another participant reflected on repeated use: “I think it was very rewarding and interesting. I believe it could give even more if one used it several times, as it becomes easier to learn how the program works, and one gets used to quite a long response time from the patient.” This statement highlights the strengths of the system's features and areas for development.

The statement that received the lowest mean score across the entire survey concerned the perceived learning benefits for participants through observing the avatar's facial and body expressions in *Crisis Support VR* (statement 5.13; mean = 2.97; Table 4). Qualitative data responses provided additional context, with several participants describing learning with *Crisis Support VR* as “a very good way to train and consequently develop.” However, some participants also emphasized that the system should be considered “a valuable complement to other learning” rather than a standalone solution. The irreplaceable value of interpersonal interaction in professional practice was frequently noted, as expressed by one participant, who stated, “I believe one shouldn't forget the importance of human-to-human interaction.” This comment emphasized that, although learning with VR-based systems offers significant benefits, it cannot fully substitute for traditional classroom-based teaching.

In addition, several participants expressed concerns about the learning environment in which the *Crisis Support VR* demonstration sessions were held. Specifically, the sessions were organized around group activities, and some participants felt that this was less conducive to individual engagement and learning. One participant remarked, “It would have been easier to make more effort if one could sit alone; there were too many people in the same room.” Another commented, “Many of us sat together and were expected to practice. This wasn't good. I would have preferred to sit alone.*”* A third participant highlighted the issue of noise interference: “An improvement would be to have fewer people in the room because they tended to talk over me and the avatar.” These responses suggest that the physical learning environment ought to be considered more thoroughly to improve both the quality of interaction with avatars and the learning experience.

## Discussion

The results of this study highlight crisis support teams’ technical openness and learning attitudes toward the GenAI-based VP system *Crisis Support VR.* Analysis of the crisis support elements of the TOLA survey data provided valuable insights into the main factors that may influence the effectiveness of integrating GenAI-based VP systems based on didactic simulation into experiential learning (13). A main finding of this study was that the statement regarding learning about serious events/humanitarian crises with the *Crisis Support VR* system received the highest mean score across the entire survey, surpassing the score for participants’ self-evaluated digital competence. This highlights the participants’ positive learning attitudes toward *Crisis Support VR* as a valuable tool that incorporates a variety of didactic simulation for learning about PFA for serious events/humanitarian crises. Another main finding of this study, aligning with the participants’ overall positive learning attitudes toward *Crisis Support VR*, was their responses about practicing communication and decision-making skills. Specifically, through listening and asking questions (verbally) in conversations with avatars. In contrast, the lowest mean score among all statements was associated with the participants’ perceived ability to interpret the avatars’ facial and bodily expressions. This highlights a critical limitation of current GenAI-based VP systems, particularly regarding their technological capacity to convey paraverbal communication and nonverbal cues.

A well-established study by Mehrabian (23) suggested that interpersonal communication consists of approximately 7% verbal content (i.e., spoken words), 38% paraverbal communication (e.g., tone of voice), and 55% nonverbal cues (e.g., emotion conveyed through facial and bodily expressions). Although these proportions should be interpreted with caution, they illustrate the substantial role of paraverbal communication and nonverbal cues in interpersonal communication, indicating that interpersonal communication is conveyed more through how something is expressed than through verbal content alone (24). When learners interact with GenAI-based VP systems, they tend to apply similar interpretative cues for interpersonal communication (25). Supporting this, Compensis (26) demonstrated that avatars capable of mimicking cues for paraverbal communication and nonverbal were perceived as more likable and trustworthy compared to avatars relaying solely on verbal content. While Kang and Lee (27) found that paraverbal communication and nonverbal cues significantly influence learner's embodiment and presence. Oh Kruzic et al. (28) emphasized that realistic facial animations, particularly when combined with bodily expressions are crucial for positive avatar interactions. Accordingly, the development of avatars capable of expressing paraverbal communication and nonverbal cues, together with technologies that can accurately track the users’ facial expressions in real time, may improve learners’ outcomes and enhance human–machine interactions. Oh Kruzic et al. (28) also notes that, within VR contexts, the use of head-mounted displays presents additional challenges for facial expression tracking. In VR based platforms such as *Crisis Support VR,* limitations in accurately conveying paraverbal communication and nonverbal cues, due to rendering, latency or head-mounted display quality may hinder learners’ ability to interpret interpersonal communication.

The findings presented in this study provide important evidence suggesting that VR platforms such as *Crisis Support VR,* should extend beyond a focus on verbal content to convey paraverbal communication and nonverbal cues in ways that learners can intuitively process. Aligning with Qiao et al. (29) and Zhang et al. (30) emphasizing that avatars can enhance engagement in certain contexts, the avatars’ limited capabilities related to conveying nuanced paraverbal communication and nonverbal cues may nonetheless reduce the perceived authenticity of interactions. Furthermore, as noted by Lukacovic et al. (15), facilitators in the context of humanitarian crises have expressed concerns regarding the perceived lack of authenticity of didactic simulations generated by GenAI-based VP systems. Learning to interact with individuals during serious events/humanitarian crises requires didactic simulations that engage learners in paraverbal communication and nonverbal cues, enabling learners to provide high-quality PFA in accordance with the five essential principles outlined by Hobfoll et al. (4).

In this context, Knowles's principles of andragogy (31) emphasize that adult learners are more engaged when they learn content that is relevant to their needs, when they are actively involved in shaping the learning process and assessment, and when new knowledge builds upon their prior experiences. *Crisis Support VR* aligns well with Knowles’ principles of andragogy because it engages learners by simulating PFA during humanitarian crises, fostering active learning and drawing on learners’ existing knowledge and experiences.

Drawing upon prior experiences requires knowledge. A hypothesis that warrants further study is whether GenAI-based VP systems involving GenAI-powered avatars, which lack visible and structured questions for learners to read and reflect on prior to verbal interaction, differ in terms of learning outcomes from VP-based systems that incorporate preprogrammed conversational components, including multiple levels of spoken words, follow-up questions, and detailed feedback structures. Simply assuming that GenAI-based VP systems are effective didactic simulation tools is hazardous, as didactic simulations clearly rely on the idea that the choice of simulation tools should be based on learners’ knowledge and experiences (10). In other words, didactic simulation tools can either facilitate or hinder the achievement of learning outcomes. In a study by Eckerström et al. (32), it was noted that preprogrammed conversational components in a VP system, absent in current GenAI-based systems, provided valuable nuances of spoken words for novice learners. Building on this, Mårtensson et al. (33) further demonstrated that these preprogrammed conversational components guided novice learners in navigating the complexities of using spoken words when asking questions during patient assessment calls.

A secondary hypothesis, aligning with Benner's (34) theory of skill acquisition from novice to expert, suggests that a VP system with preprogrammed conversational components may be less important for expert learners who are more likely to rely on their accumulated knowledge and clinical experiences. As Dewey (11) emphasized, reflective practice is not merely a rational intellectual process but an action rooted in understanding the meaning of one's experiences. This suggests that for expert learners, the value of VP-systems may lie more in their ability to facilitate reflection and contextual application than in their structured, preprogrammed conversational components. A notable finding of this study was that participants were more skeptical about *Crisis Support VR's* ability to support deeper learning for serious events/humanitarian crises when compared to traditional classroom-based teaching, specifically human-to-human interactions between a facilitator(s) and learners. This finding reinforces the argument that GenAI-based VP systems should be used to facilitate, rather than replace, interpersonal interactions in professional learning contexts.

Furthermore, the findings indicate that learners expect more from facilitators than technical proficiency with the system and subject expertise alone. This has important implications for facilitators involved in didactic simulations using GenAI-based VP systems. Facilitating learners also requires an understanding of how to manage the physical learning environment to ensure that the immersive qualities of VR interactions are not compromised. Factors such as large group sizes and background noise, which can overpower or drown out the avatar communication within the VP system, may significantly hinder learning and diminish learners’ overall immersion in the simulation experience. Nevertheless, another main finding of this study was that the statement regarding participants’ willingness to continue practicing PFA with the *Crisis Support VR* system received among the highest mean score across the entire survey. This is a highly encouraging result that supports further implementation, evaluation and generalizability of the system, as it suggests that *Crisis Support VR* may be a valuable didactic simulation tool for helping crisis support teams learn PFA that aligns with the five essential principles outlined by Hobfoll et al. (4).

However, several limitations should be acknowledged. First, the TOLA survey was designed as an exploratory instrument to describe participants’ technological openness and learning attitudes toward using digital technology, rather than as a fully validated psychometric scale. Therefore, the results of the five-point Likert scale statements should be interpreted descriptively. Additionally, while the study focused on technical openness and learning attitudes, actual learning outcomes were not assessed. Future research should evaluate the effectiveness of GenAI-based VP systems, such as *Crisis Support VR*, in facilitating measurable learning outcomes. Second, the study was conducted at a single site in western Sweden with a relatively highly educated sample, which may limit the generalizability of the findings. The study also involved a relatively small sample, making it vulnerable to participant dropout. All participants in this study were familiar with the *Crisis Support VR* system, which may have introduced response bias. Furthermore, the demonstration sessions were conducted by a project leader who is also a co-author, and participants were recruited within the same institutional context. These factors may have influenced responses through social desirability or institutional loyalty, potentially contributing to the generally high mean scores and affecting the accuracy of the self-reported data. Furthermore, the physical and environmental setup may have affected participants’ responses more than the system itself, including technical constraints such as rendering, latency, or limitations of head-mounted displays. These factors should be considered when interpreting the findings. Future research could address this limitation by conducting multisite (e.g., national and/or global) studies to capture a wider range of contexts and enhance external validity.

## Conclusion

Against the backdrop of escalating global humanitarian crises and the urgent need to strengthen awareness of and support for humanitarian action, *Crisis Support VR* represents an innovative didactic simulation tool for learning PFA, serving as a valuable complement to traditional classroom-based didactics. As the study is limited to self-reported perceptions, no conclusions can be drawn regarding learning outcomes or actual performance. However, the findings indicate that *Crisis Support VR* was perceived as a meaningful didactic simulation tool for learning PFA, suggesting its potential to support PFA training for crisis support teams. Future research should assess whether *Crisis Support VR* translates into measurable learning outcomes and, in doing so, contributes to preparedness and response capacity during humanitarian crises.

## Data Availability

The raw data supporting the conclusions of this article will be made available by the authors, without undue reservation.
