# Supplementary material for: Crisis support teams’ technological openness and learning attitudes toward the AI based virtual patient system crisis support VR
Source: Front Digit Health. 2026 Jun 9;8:1805454. doi: 10.3389/fdgth.2026.1805454 (PMC13288512; doi:10.3389/fdgth.2026.1805454)
Supplement: Supplementary file 1 [file Datasheet1.pdf]

UNIVERSITY
OF SKÖVDE

A survey of technological openness and learning attitudes (TOLA) toward using digital technology

$

Demographic characteristics

Sex: Male □ Female □ Do not want to declare □

Age: 18–25 □ 26–30 □ 31–35 □ 36–40 □ 41–45 □ 50–55 □ 56–60 □ 61–65 □ > 65 □

Profession: ____

Years working in the profession: ____

Do you do clinical work and see patients?: Yes □ No □

Do you have a management role?: Yes □ No □

UNIVERSITY OF SKÖVDE

The following section presents statements related to digital competence.

1. To what extent do the following statements apply to you, with 1 indicating "Not at all true" and 5 indicating "Completely true."

	1	2	3	4	5
**1.1. I have good digital competence.**	☐	☐	☐	☐	☐
**1.2. It is important for me to use the latest digital technology.**	☐	☐	☐	☐	☐
**1.3. I find it easy to understand instructions about using digital technology.**	☐	☐	☐	☐	☐
**1.4. I have sufficient digital competence to ensure my own learning.**	☐	☐	☐	☐	☐
**1.5. I have sufficient digital competence to teach others how to use digital technology.**	☐	☐	☐	☐	☐

$

The following section presents statements related to GenAI systems, such as ChatGPT, Stable Diffusion, and CoPilot, which are systems that generate text and/or images.

2. To what extent do the following statements apply to you, with 1 indicating "Not at all true" and 5 indicating "Completely true."

	1	2	3	4	5
**2.1. In my private life, I use GenAI systems every week (i.e., for chatting, text, images, sound, and games).**	☐	☐	☐	☐	☐
**2.2. I believe that GenAI systems manage and store data safely.**	☐	☐	☐	☐	☐
**2.3. I trust the accuracy and reliability of the content provided by GenAI systems.**	☐	☐	☐	☐	☐
**2.4. I expect that GenAI systems will help me acquire new knowledge and/or deepen my existing knowledge.**	☐	☐	☐	☐	☐

$

The following section presents statements related to GenAI systems, such as chatbots, and interactive tablets for use in primary care.

3. To what extent do the following statements apply to you, with 1 indicating "Not at all true" and 5 indicating "Completely true."

	1	2	3	4	5
**3.1. I feel positive about the use of GenAI systems in health care.**	☐	☐	☐	☐	☐
**3.2. I expect that GenAI systems will help me develop my critical thinking and reasoning skills.**	☐	☐	☐	☐	☐

**3.3. I expect that GenAI systems will help me listen to and understand patients.**	☐	☐	☐	☐	☐
**3.4. I expect that GenAI systems will help me read and interpret patients' facial expressions and body language.**	☐	☐	☐	☐	☐
**3.5. I expect that GenAI systems will help me ask patients relevant questions.**	☐	☐	☐	☐	☐
**3.6. I expect that GenAI systems will be safe for conducting <u>health assessments</u> (i.e., ensuring patient safety).**	☐	☐	☐	☐	☐
**3.7. I expect that GenAI systems will be safe for conducting <u>diagnoses</u> (i.e., ensuring patient safety).**	☐	☐	☐	☐	☐
**3.8. I feel positive about the use of GenAI systems for medical record documentation purposes (i.e., ensuring patient safety).**	☐	☐	☐	☐	☐
**3.9. I expect that GenAI systems will complement health-care professionals in recommending health measures (e.g., lifestyle changes regarding smoking, diet, sleep, and exercise).**	☐	☐	☐	☐	☐
**3.10. I expect that GenAI systems will replace health-care professionals in recommending health measures (e.g., lifestyle changes regarding smoking, diet, sleep, and exercise).**	☐	☐	☐	☐	☐
**3.11. I perceive a risk that GenAI systems will replace in-person patient consultations.**	☐	☐	☐	☐	☐

The following section presents statements related to <u>GenAI-based virtual patient (VP) systems</u> in health-care and health-care education contexts (i.e., systems that can, for example, simulate interactions with patients in terms of communication, behaviors, and illness).

4. To what extent do the following statements apply to you, with 1 indicating "<u>Not at all true</u>" and 5 indicating "<u>Completely true</u>."

	1	2	3	4	5
**4.1. I feel positive that GenAI-based VP systems can help me practice in various health-care contexts.**	☐	☐	☐	☐	☐
**4.2. I expect that GenAI-based VP systems will allow me to practice following patients through various health-care chains.**	☐	☐	☐	☐	☐
**4.3. I believe that GenAI-based VP systems will be effective tools for increasing awareness of one's own assumptions and biases toward patients.**	☐	☐	☐	☐	☐
**4.4. I expect that GenAI-based VP systems will help me develop my critical thinking and reasoning skills.**	☐	☐	☐	☐	☐
**4.5. I expect that GenAI-based VP systems will help me practice using various assessment and rating scales.**	☐	☐	☐	☐	☐
**4.6. I expect that GenAI-based VP systems will be safe for conducting <u>health assessments (</u>i.e., ensuring patient safety).**	☐	☐	☐	☐	☐
**4.9. I expect that GenAI-based VP systems will be safe for conducting <u>diagnoses</u> (i.e., ensuring patient safety).**	☐	☐	☐	☐	☐
**4.10. I expect that GenAI-based VP systems will provide opportunities to practice implementing patient-related actions (e.g., making referrals to other professionals or external agencies).**	☐	☐	☐	☐	☐
**4.11. I expect that GenAI-based VP systems will help me listen to and understand patients.**	☐	☐	☐	☐	☐
**4.12. I expect that GenAI-based VP systems will help me read and interpret patients' facial expressions and body language.**	☐	☐	☐	☐	☐
**4.13. I expect that GenAI-based VP systems will help me ask patients relevant questions.**	☐	☐	☐	☐	☐
**4.14.I expect that GenAI-based VP systems will give me clear recommendations and instructions on what I need to practice.**	☐	☐	☐	☐	☐
**4.15. I perceive a risk that GenAI-based VP systems will replace classroom teaching (e.g., meetings between educators and learners).**	☐	☐	☐	☐	☐
**4.16. I believe that GenAI-based VP systems manage and store data safely.**	☐	☐	☐	☐	☐
**4.17. I believe that GenAI-based VP systems will contribute to my development as a more professional and competent caregiver.**	☐	☐	☐	☐	☐

4.18. Have you previously used generative AI-based VP systems for educational purposes?
Yes □ No □
If yes, please specify which system(s) you used and describe the educational context?

4.19. Free-text response: What factors do you believe promote learning when using generative AI-based VP systems in health-care and health-care education contexts?

4.20. Free-text response: What factors do you believe hinder learning when using generative AI-based VP systems in health-care and health-care education contexts?

4.21. Free-text response: Would you like to share any additional thoughts about how you perceive generative AI-based VP systems in health-care and health-care education contexts?

The following section is only for those of you who practiced using the GenAI-based VP system Crises Support VR.

5. To what extent do the following statements apply to you, with 1 indicating "Not at all true" and 5 indicating "Completely true."

	1	2	3	4	5
**5.1. I am positive about serious events/humanitarian crises with the GenAI based VP system Crisis Support VR.**	☐	☐	☐	☐	☐
**5.2. I believe that Crisis Support VR is a good complement to practice psychological first aid.**	☐	☐	☐	☐	☐
**5.3. I expect that I can learn more about serious events/humanitarian crises with Crisis Support VR than through traditional classroom teaching (i.e., meetings between educators and learners).**	☐	☐	☐	☐	☐
**5.4. I want to continue practicing psychological first aid with Crisis Support VR.**	☐	☐	☐	☐	☐
**5.5. I believe that the digital technology in the Crisis Support VR system worked well.**	☐	☐	☐	☐	☐
**5.6. I believe it was easy to understand the instructions about navigating Crisis Support VR.**	☐	☐	☐	☐	☐
**5.7. I believe it was easy to navigate Crisis Support VR.**	☐	☐	☐	☐	☐
**5.8. I believe that Crisis Support VR represented a variety of crisis situations (i.e., terrorist attacks, wildfires, floods, etc.).**	☐	☐	☐	☐	☐
**5.9. I believe that Crisis Support VR reflects a norm-critical perspective, meaning that it is designed to minimize built-in biases.**	☐	☐	☐	☐	☐
**5.10. Crisis Support VR gave me relevant feedback on my performance.**	☐	☐	☐	☐	☐
**5.11. I felt safe when I practiced psychological first aid with Crisis Support VR.**	☐	☐	☐	☐	☐
**5.12. I believe that in Crisis Support VR I could practice listening to the avatar.**	☐	☐	☐	☐	☐
**5.13. I believe that in Crisis Support VR I could practice seeing the avatar's facial and body expressions.**	☐	☐	☐	☐	☐
**5.14. I believe that in Crisis Support VR I could practice asking questions to the avatar.**	☐	☐	☐	☐	☐

5.15. Free-text response: Would you like to share any additional thoughts about how you perceive your learning through the use of Crisis Support VR?

